# Influence of Cutting Styles on Antioxidant Capabilities of Fresh-Cut Cauliflower by Regulating ROS Metabolism and Antioxidant Enzyme Activity

**DOI:** 10.3390/antiox14101188

**Published:** 2025-09-28

**Authors:** Qihan Guo, Bingheng Li, Jiarui Wang, Minke Shi, Jiayu Wang, Yan Chen, Yunjie Zhang, Sarengaowa, Ying Xiao, Ke Feng

**Affiliations:** 1 Faculty of Chinese Medicine, Macau University of Science and Technology, Macao 999078, China; 2 Medical Sciences Division, Macau University of Science and Technology, Macao 999078, China; 3 College of Life Science, Zhuhai College of Science and Technology, Zhuhai 519041, China

**Keywords:** fresh-cut cauliflower, cutting styles, antioxidant capabilities, ROS metabolism, antioxidant enzyme activity

## Abstract

The enzymatic browning and oxidative deterioration of cauliflower during mechanical processing are major challenges for the fresh-cut cauliflower industry. The primary objective of this study was to investigate the impact of cutting styles on the antioxidant capacity of fresh-cut cauliflower during storage. One flower (12 × 1.8 cm) of cauliflower was designated as cutting style 1 (CS1). CS1 was cut longitudinally into strips as cutting style 2 (CS2). CS1 was also cut transversely into cubes, as cutting style 3 (CS3), and longitudinally and transversely into small cubes, as cutting style 4 (CS4). Results indicated that at the conclusion of the 72 h storage period, cutting treatments enhanced the total antioxidant capacity of fresh-cut cauliflower in the ABTS assay by 128.1%, 82.9%, 50.1%, and 38.9% for CS1, CS2, CS3, and CS4, respectively. All treatment groups except CS1 exhibited increased total antioxidant capacity in the FRAP assay. Phenolic compound accumulation increased by 106.82%, 105.24%, 270.4%, and 295.3% in CS1, CS2, CS3, and CS4, respectively. In addition, the O_2_^−^· scavenging activity was enhanced; the activities of antioxidant-related enzymes, including catalase (CAT) and superoxide dismutase (SOD), were also increased. In conclusion, the extent of the effect on antioxidant capacity was as follows: CS4 > CS3 > CS2 > CS1. This study has elucidated the patterns of influence exerted by cutting methods upon the quality of fresh-cut cauliflower, thereby providing theoretical foundations and empirical data to inform the selection of appropriate cutting techniques for both commercial processing and domestic culinary applications.

## 1. Introduction

Cauliflower (*Brassica oleracea* L. var. *botrytis* L.) is a globally significant Brassicaceous vegetable, renowned for its high content of vitamin C, phenolic compounds, and glucosinolates [1,2]. As the world’s largest producer, China has a substantial interest in optimizing the postharvest value of this crop [3]. Fresh-cut vegetables trigger a series of physiological and biochemical reactions, including accelerated nutrient loss, tissue browning, and microbial invasion, while its antioxidant capacity is improved [2,4]. This is due to the fact that mechanical damage during processing activates two types of plant defense mechanisms [4]; the plant’s oxidative enzyme system is activated, with enzymes such as superoxide dismutase (SOD), peroxidase (POD), and catalase (CAT) scavenging reactive oxygen species (ROS) through the ascorbate–glutathione (AsA-GSH) cycle to maintain cellular homeostasis [5]. The phenylalanine metabolic pathway is activated, with phenolic synthesis catalyzed by phenylalanine ammonia-lyase (PAL) and cinnamic acid 4-hydroxylase (C4H), facilitating wound repair [5,6]. Rupture of the cell membrane leads to the oxidation of phenolics to quinones, which are catalyzed by polyphenol oxidase (PPO) to form melanin polymers that trigger browning [7]. These phenomena have also been observed in studies on fresh-cut cauliflower [8].

In recent years, significant progress has been made in studying the effects of different cutting methods on the quality of fresh-cut fruits and vegetables. In fresh-cut potatoes, Hu et al. (2021) found that cutting-induced increases in lipoxygenase, polyphenol oxidase, and peroxidase activity led to the accumulation of malondialdehyde and browning, with the extent of impact being slice > strip > cube [9]. Similarly, studies on fresh-cut kiwifruit have shown that total phenolic content and DPPH radical scavenging activity significantly increase with increasing wound intensity [10]. Similar results have also been observed in sweet potatoes and cucumbers [11,12]. Current research suggests that mechanical damage incurred during the fresh-cut processing of cauliflower triggers enzyme-catalyzed oxidative browning and deterioration in quality, ultimately leading to a decline in the quality of fresh-cut cauliflower [8,13].

Few studies have examined the effect of cutting styles on regulating ROS synthesis and antioxidant enzyme activity in fresh-cut cauliflower. Further studies should focus on evaluating the wounding effects of appropriate cutting practices on the commercialization of fresh-cut cauliflower, taking into account the associated changes in ROS and antioxidant enzyme activities during storage. This study analyzed the effects of different cutting styles on the antioxidants and enzymatic activity, as well as the ROS and antioxidant capacity, of fresh-cut cauliflower. The findings aim to provide a scientific basis for selecting the best cutting method to maximize health-promoting benefits while maintaining the commercial quality of fresh-cut cauliflower.

## 2. Materials and Methods

### 2.1. Materials and Treatments

**Figure 1:**
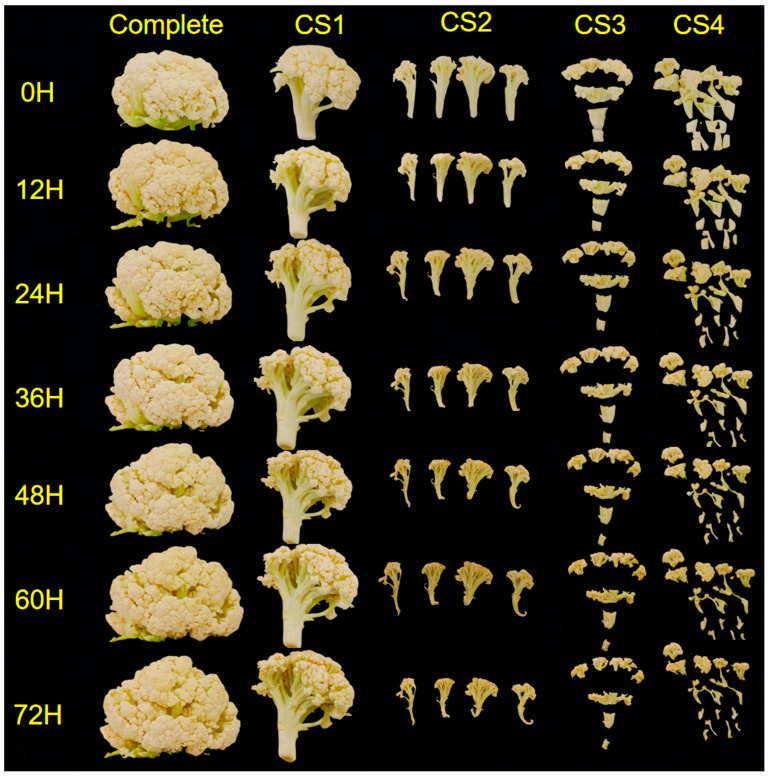
The changes in fresh-cut cauliflower in different treatment groups during storage period. Among them, Complete is a whole cauliflower. CS represents cutting style. The one flower of cauliflower was cut as CS1 (cutting styles1). The CS1 was cut longitudinally into strips as CS2 (cutting styles 2). The CS1 was cut transversally into cubes as CS3 (cutting styles3). The CS1 was cut simultaneously longitudinally and transversally into small cubes as CS4 (cutting styles 4).

**Figure 2:**
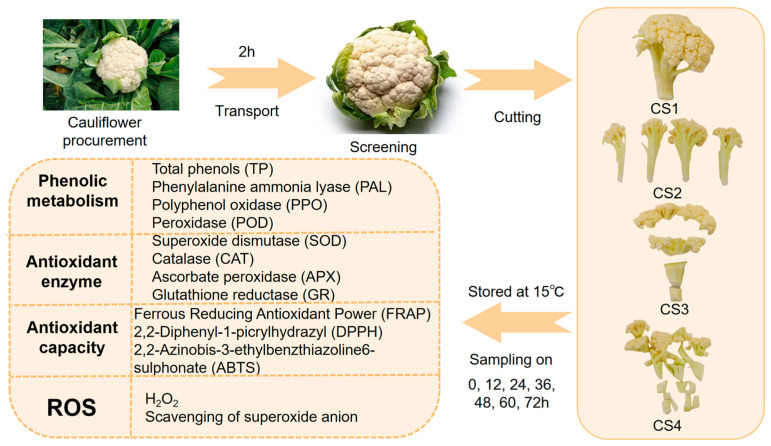
Experimental workflow of the antioxidant capabilities of fresh-cut cauliflower with different cutting styles. CS represents cutting style. The one flower of cauliflower was cut as CS1 (cutting styles1). The CS1 was cut longitudinally into strips as CS2 (cutting styles 2). The CS1 was cut transversally into cubes as CS3 (cutting styles3). The CS1 was cut simultaneously longitudinally and transversally into small cubes as CS4 (cutting styles 4).

Cauliflower was purchased from Hengqin Jiarong Supermarket (Legendale Pangdu Plaza Store) in Zhuhai, and the variety was pine cauliflower. Select fresh cauliflowers of uniform specification (diameter 28 ± 3 cm), consistent coloration, and free from disease, insect damage, mechanical injury, or decay. Harvested specimens must be transported to the laboratory within one hour. Ensure colour uniformity by selecting plants with no visual defects (such as yellowing or browning) and compact, pure-white heads. Selected cauliflower plants were sterilized with 0.02% sodium hypochlorite solution (Macklin, Shanghai, China), washed with distilled water, and then placed on the table to dry naturally. The length of the cutting style 1 (CS1) cauliflower root incision to the tip of the corolla was approximately 12 cm, the weight of the tissue was 50 g, and the diameter of the wound surface was 1.8 cm. CS1 was cut into 4 pieces along the stem longitudinally to obtain cutting style 2 (CS2). The CS1 was then cut transversally into 4 cubes, resulting in cutting style 3 (CS3). CS1 was cut into 16 pieces, simultaneously along both longitudinal and transverse directions, into small cubes, resulting in cutting style 4 (CS4) (Figure 1). Cauliflower was randomly assigned to each cutting and CS1 was used as a control. The experimental workflow of fresh-cut cauliflower is shown in Figure 2. This study utilized 36 cauliflower heads (biological replicates), all sourced from the same batch to minimize variability. Each cutting method (CS1–CS4) was assigned three biological replicates (independent cauliflower heads), with each biological replicate undergoing triplicate analysis to validate the reproducibility of results.

Cauliflower samples were placed in polypropylene containers (thickness: 110 μm; size: 11.5 cm × 9.5 cm × 6.2 cm; porosity), with each container holding 120 g of sample. The containers were then wrapped in polyethylene film (thickness: 6 μm; size: 30 cm × 25 cm), and stored at a temperature 59 ± 3.6 °F (15 ± 2 °C) (although this is not carried out in the fresh-cut cauliflower industry, it is carried out to speed up the wound stress response) [14], collected intact, and differentially treated at 12-hourly intervals (72 h deadline), frozen in liquid nitrogen, and then frozen in a cryogenic pulverizer by 5000 (r/min) (JFC-2000, Nippon Analytical Industries Inc., Tokyo, Japan) to obtain cauliflower powder for analysis of phenol metabolism, antioxidants enzymatic activity, ROS and antioxidant capacity.

### 2.2. Total Phenol (TP) Content and Phenylalanine Ammonia-Lyase (PAL) Activity Measurement

Total phenol (TP) content and phenylalanine ammonia-lyase (PAL) activity were determined using commercially available kits (Suzhou Comin Biotechnology Co., Ltd., Suzhou, China). TP content a total of 0.01 g of dried cauliflower sample powder was taken from each group, and we followed the instructions in the reagent kit manual. Measurements were performed at 760 nm using a SpectraMax^®^ iD3 microplate reader (Molecular Devices, LLC, San Jose, CA, USA). The results were expressed as mg GAE/g D. PAL activity a total of 0.5 g of frozen cauliflower sample powder was taken for each group, and the instructions in the reagent kit manual were followed. The sample was assayed at 290 nm using a SpectraMax^®^ iD3, and the results were expressed as U/g.

### 2.3. Polyphenol Oxidase (PPO) and Peroxidase (POD) Activity Measurement

The activities of PPO and POD were determined using commercially available kits (Suzhou Comin Biotechnology Co., Ltd., Suzhou, China). Refer to the PPO activity test kit manual, for each group 0.5 g of frozen cauliflower sample powder was used [15]. The activity was determined using commercially available kits, following the instructions in the reagent kit manual. The samples were assayed at 525 nm using a SpectraMax^®^ iD3, and the results were expressed as U/g. POD assay testing for each group, 0.5 g of frozen cauliflower sample powder was taken, and the instructions in the reagent kit manual were followed. The sample was assayed at 470 nm using a SpectraMax^®^ iD3, and the results were expressed as U/g.

### 2.4. Reactive Oxygen Species (ROS) Measurement

The scavenging activity of H_2_O_2_ and superoxide anion radicals (O_2_^−^·) was determined using commercially available kits (Suzhou Comin Biotechnology Co., Ltd., Suzhou, China). Refer to the H_2_O_2_ test kit manual, for each group, 0.1 g of frozen cauliflower sample powder was taken, and the instructions in the reagent kit manual were followed. The sample was assayed at 415 nm using a SpectraMax^®^ iD3, and the results were expressed as μmol/g. Refer to the O_2_^−^· test kit manual, for each group, 0.1 g of frozen cauliflower sample powder was taken, and the instructions in the reagent kit manual were followed. The sample was assayed at 530 nm using a SpectraMax^®^ iD3, and the results are expressed as a percentage.

### 2.5. Enzymes Activity Measurement

#### 2.5.1. Superoxide Dismutase (SOD) and Catalase (CAT) Activity Measurement

The activities of SOD and CAT were determined using commercially available kits (Suzhou Comin Biotechnology Co., Ltd., Suzhou, China). The activities of SOD and CAT, for each group 0.1 g of frozen cauliflower sample powder was taken, and the instructions in the reagent kit manual were followed. The sample of SOD was assayed at 560 nm/CAT was assayed at 405 nm using a SpectraMax^®^ iD3, and the results were expressed as U/g.

#### 2.5.2. Glutathione Reductase (GR) and Ascorbate Peroxidase (APX) Activity

GR and APX activities were determined using commercially available kits (Suzhou Comin Biotechnology Co., Ltd., Suzhou, China). The activities of GR, for each group 0.1 g of frozen cauliflower sample powder was taken, and the instructions in the reagent kit manual were followed. The rate of decrease in absorbance at 340 nm for 3 min of measured using ultraviolet–visible spectroscopy (UV-Vis spectra). These spectra were recorded with a Shimadzu UV-2600i spectrophotometer (Shimadzu Corporation, Kyoto, Japan). The results of the experiments to calculate the activity of GR were expressed as nmol/min/g. APX activities, for each group 0.1 g of frozen cauliflower sample powder was taken, and the instructions in the reagent kit manual were followed. The rate of oxidation of measured using a Shimadzu UV-2600i at 290 nm for 2 min to calculate the activity of APX. The results were expressed as nmol/min/g.

### 2.6. Antioxidant Capabilities Measurement

DPPH, ABTS, and FRAP were determined using commercially available kits (Suzhou Comin Biotechnology Co., Ltd., Suzhou, China). DPPH was determined according to the DPPH free radical scavenging method. For DPPH, ABTS, and FRAP assays, 0.5 g of the frozen cauliflower sample powder was used, and the instructions in the reagent kit manual were followed. The changes in absorbance were measured using a SpectraMax^®^ iD3 reader, and the results are expressed in terms of μmol Trolox equivalent per gram of fresh tissue (μmol Trolox/g).

### 2.7. Cauliflower Weight Loss and Color Measurement

Samples (120 g) were placed polypropylene containers. Samples were weighed using a digital balance (BSA224S-CW, Sartorius, Göttingen, Germany) during storage. The weight loss rate equation was evaluated [16]:

Weight loss rate (%) = [(m_1_ − m_2_)/m_1_] × 100

where m_1_ is the initial weight (in g) and m_2_ is the weight at the specified time point (in g).

Color parameters of cauliflower, including L* (lightness), a* (+a* = redness, −a* = greenness), and b* (+b* = yellowness, −b* = blueness), were evaluated using a NR10QC colorimeter (3nh, Shenzhen, China). The experiments were performed in triplicate. In total, 24 caskets of samples (6 caskets per treatment group) were measured. The color parameter values for each treatment group were obtained by averaging 6 assays in the central region of the surface. Measurements of spectral reflectance were performed directly onto the vegetables surface (viewingt area = 10 × 10 mm). To characterize more precisely the color of cauliflowers, the hue angle (hue = arctan(b*/a*) if a* > 0 and hue = arctan(b*/a*) + 180 if a* < 0) was determined to indicate color changes between a* (green color) and the intersection of a* and b*, from green (hue = 0) to yellow color (hue = 90) [16,17].

### 2.8. Sensory Analysis

After storing the samples at 4 °C for 8 days, sensory characteristics of each treatment group were evaluated by regular cauliflower consumers. A total of 20 participants (10 females and 10 males, aged between 22 and 45 years) were recruited from students and staff at the University of Science and Technology of Macau (Macao, China). At the start of the experiment, they received training to familiarize themselves with the characteristics of fresh-cut cauliflower. Sensory evaluations were conducted in an independent compartment of the sensory laboratory. The order of sample presentation was randomly and evenly distributed among the evaluators. Evaluators scored the color, appearance, odor, texture, and overall acceptability of fresh-cut cauliflower at room temperature (approximately 25 °C) using a 9-point liking scale, which ranged from 1 (very dislike) to 9 (very like). The pleasantness rating scale is as follows: 9 points, very liked; 8 points, liked; 7 points, moderately liked; 6 points, slightly liked; 5 points, neutral; 4 points, slightly disliked; 3 points, moderately disliked; 2 points, disliked; 1 point, very disliked [16,18]. The average response value for each attribute was calculated, samples with scores below 5 were considered unacceptable [16,17,19].

### 2.9. Statistics

All measurements were performed in triplicate to complete. All data were analyzed using GraphPad Prism version 10.4.0 for Windows (GraphPad Software, Boston, MA, USA). One-way analysis of variance (ANOVA) and the least significant difference (LSD) method (*p* < 0.05) were employed to determine the intergroup significance of differences between groups. Correlations were also analyzed using Pearson’s correlation (two-tailed test) for different indicators, and the data were plotted.

## 3. Results

### 3.1. Total Phenol (TP) Content and Phenylalanine Ammonia-Lyase (PAL) Activity

**Figure 3:**
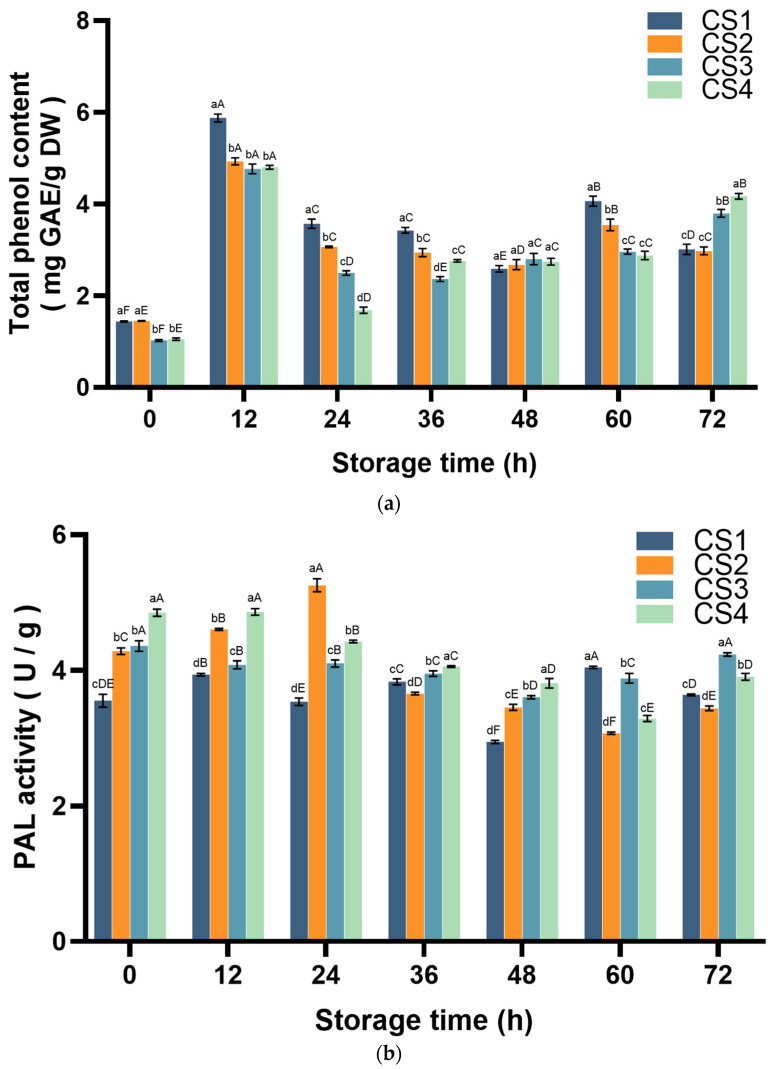
Effect of cutting styles on total phenolic content (**a**) and PAL activity (**b**) of fresh-cut cauliflower. Columns with vertical bars represent mean SD values (*n* = 3). Values with different letters are significantly different at *p* < 0.05. Lowercase letters represent significant differences between treatment factors; uppercase letters represent significant differences between storage times. CS is cutting styles. The cauliflower flower was cut as CS1 (cutting style 1). The CS1 was cut longitudinally into strips, resulting in CS2 (cutting styles 2). CS1 was cut transversely into cubes, similar to CS3 (cutting style 3). CS1 was then cut simultaneously, both longitudinally and transversally, into small cubes, resulting in CS4 (cutting style 4).

TP was determined using the Folin–Ciocalteu method [20]. TP substances are highly efficient antioxidants of plant origin and are widely present as secondary metabolites in fruits and vegetables. More than 6500 kinds of phenolic substances and their derivatives have been identified, constituting the most important source of antioxidants in the human diet [21,22,23]. The variation in TP content of fresh-cut cauliflower over time is shown in Figure 3a. In the initial 12 h stress response, all treatments showed a significant increase (*p* < 0.05), with the TP level in the minimum injury CS1 group reaching its peak, increasing by 308.4% compared to 0 h. Subsequently, CS1 and CS2 exhibited a rapid decline within 12–48 h, followed by a brief recovery at 60 h. CS3 and CS4 maintained continuous accumulation after 24 h. At 72 h, they increased by 270.4% (CS3) and 295.3% (CS4), respectively, compared with 0 h, while the TP contents of CS1 and CS2 increased by 106.82% and 105.24%, respectively, compared with 0 h. At 72 h, TP was higher in CS3 and CS4 than in CS1 and CS2.

The PAL enzyme, as a rate-limiting enzyme, plays a key role in the metabolism of phenylpropenoids. It is often used as an important indicator of plant stress resistance and regulates the biosynthesis of phenolic antioxidants in fresh-cut cauliflower [24,25,26]. PAL was determined by measuring the rate of increase in PAL-catalyzed cleavage of L-phenylalanine to trans-cinnamic acid (CA) [27]. The variation in PAL activity over time is illustrated in Figure 3b. CS1 changed slightly, reaching its lowest point at 48 h (a 17% decrease from 0 h) and then reaching its highest point at 60 h (a 13.8% increase from 0 h). CS2 exhibited an upward trend in the first 24 h, reaching its peak at 24 h (a 22.7% increase compared to 0 h), and then continuously declined before recovering at 72 h. CS3 decreased slightly during storage but returned to the same level at the beginning and end of the storage period (*p* < 0.05) and exhibited higher activity than the other groups. After maintaining high activity at the beginning, CS4 activity decreased slowly and then slightly rebounded at 72 h.

### 3.2. Polyphenol Oxidase (PPO) and Peroxidase (POD) Activity

**Figure 4:**
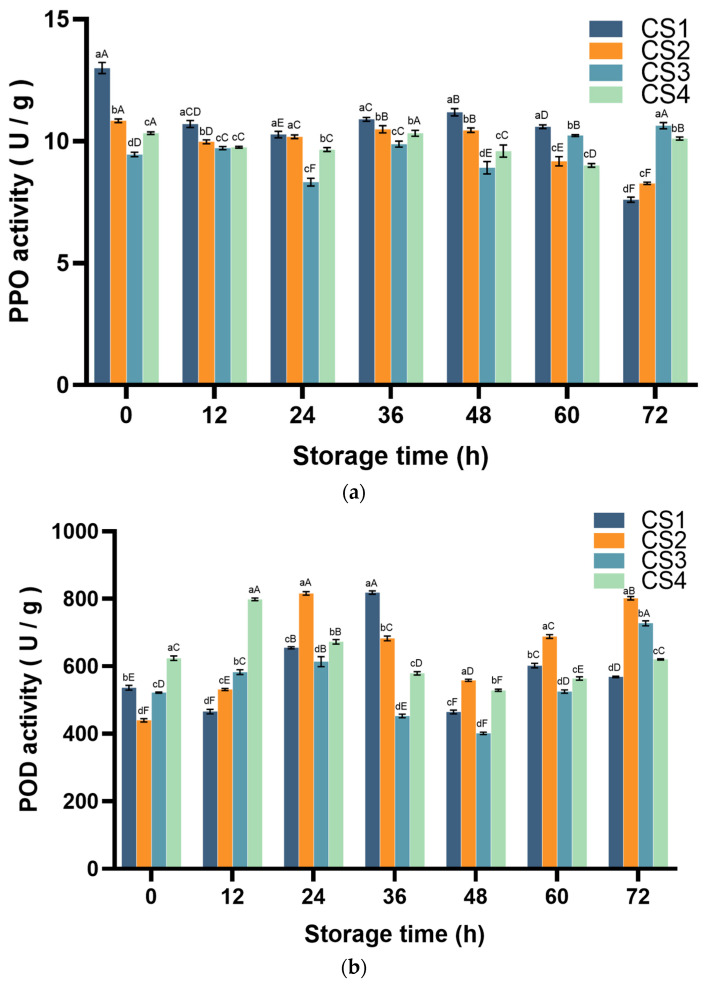
Effect of cutting method on PPO (**a**) and POD (**b**) activity of cauliflower. Columns with vertical bars represent mean SD values (*n* = 3). Values with different letters are significantly different at *p* < 0.05. Lowercase letters represent significant differences between treatment factors; uppercase letters represent significant differences between storage times.

PPO and POD, as browning-related enzymes, can oxidize the browning substrate to quinone and reduce the oxidation rate to a certain extent [28]. The principle of the PPO assay is based on the ability of PPO to catalyze the oxidation of catechols to quinones. The changes in PPO activity are shown in Figure 4a. The variation pattern of CS1/CS2 is as follows: it exhibits a wavy attenuation in the first 48 h (all rose at 48 h and then declined) and reaches its lowest activity in the last 72 h (CS1 and CS2 decrease by 41.5% and 23.7%, respectively, compared to 0 h). The activity of CS3 increased in a wave pattern, and after a slight increase at 12 h, it decreased at 24 h (*p* < 0.05). It rose at 36 h, then decreased at 48 h, and then gradually increased, peaking at 72 h, and finally increased by 12.4% compared with the initial time. After starting the 12 h decline, CS4 rebounded to the initial level at 36 h (*p* < 0.05), then decreased slightly and rose again at 72 h. The changes in POD activity are illustrated in Figure 4b. In the first 36 h, activity increased once in each treatment group. CS4 first reached its peak at 12 h, increasing by 28.0% compared to 0 h. CS2 and CS3 increased by 85.6% and 17.6%, respectively, at 24 h compared to 0 h. CS1 increased by 52.7% at 36 h compared to 0 h. After that, the activity of POD in each group began to decline rapidly and then rose again after the end of storage. The POD assay is based on the ability of POD to catalyze the oxidation of guaiacol by H_2_O_2_ to produce a reddish brown product, which has characteristic light absorption at 470 nm, and the peroxidase activity can be determined by the change in absorbance value at 470 nm [15]. CS1 continued to rise after a 12 h decline, peaking at 36 h. Both CS2 and CS3 increased during 0–24 h, decreased during 24–48 h, and then rose again from 48 to 72 h. The POD activity of CS2 remained consistently higher than that of CS3 after 24 h. CS4 peaked at 12 h, then continued to decline and rose after 48 h. At 72 h, the POD activities of CS2 and CS3 were significantly increased by 43.6% and 81.5%, respectively, compared with that at 0 h, whereas the CS1 and CS4 POD activities were significantly increased by 22.4% and 17.3% at 72 h compared to 0 h for CS1 and CS4, respectively.

### 3.3. Reactive Oxygen Species (ROS)

**Figure 5:**
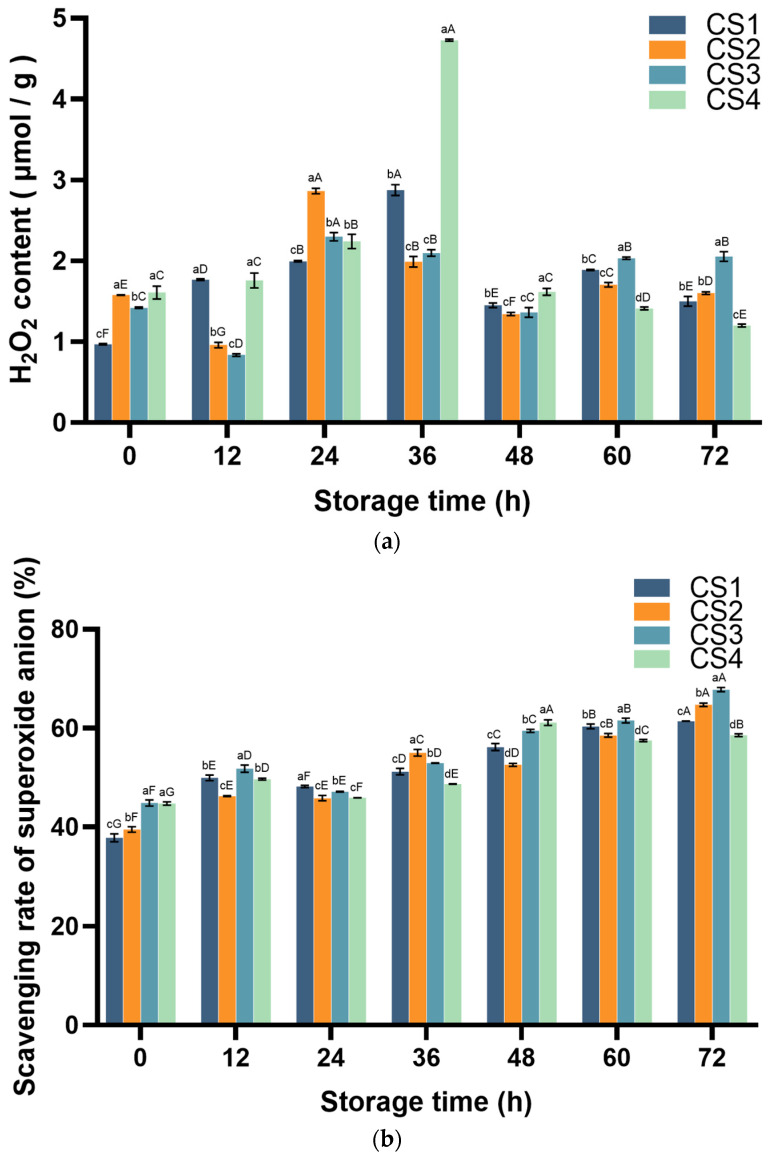
Effect of cutting method on H_2_O_2_ content (**a**) and scavenging of superoxide anion (**b**) of cauliflower. Columns with vertical bars represent mean SD values (*n* = 3). Values with different letters are significantly different at *p* < 0.05. Lowercase letters represent significant differences between treatment factors; uppercase letters represent significant differences between storage times.

The determination of H_2_O_2_ was based on the formation of a yellow titanium peroxide complex between H_2_O_2_ and titanium sulfate with a characteristic absorption peak at 415 nm, the content of H_2_O_2_ was obtained by measuring the change at 415 nm [29]. The determination of O_2_^−^· scavenging ability was based on the superoxide anion generated by the AP-TEMED system formed by ammonium persulfate (AP) and N,N,N′,N′-Tetramethylethylenediamine (TEMED), which reacted with hydroxylamine hydrochloride to form NO_2_^−^, and NO_2_^−^ reacted with p-aminobenzenesulfonic acid and α-naphthylamine to form red azo compounds, with a characteristic absorption peak of 530 nm, and the scavenging ability of the samples for superoxide anion showed a negative correlation with an absorbance value of 530 nm [30]. The changes in hydrogen peroxide (H_2_O_2_) accumulation and O_2_^−^· scavenging activity are illustrated in Figure 5. For the H_2_O_2_ content, CS1/CS4 continuously increased from 36 h (increasing by 196.9% and 194.1%, respectively, compared with 0 h). At 48 h, a sharp decline occurred simultaneously (CS1 and CS4 decreased by 49.6% and 65.8%, respectively). After 72 h, CS1 increased by 55.1% compared to 0 h, while CS4 decreased by 25.4% compared to 0 h. For CS2/CS3, after a short-term decrease at 12 h (decreasing by 39.2% and 41.1%, respectively, compared to 0 h), a rapid increase occurred at 24 h (increasing by 198.6% and 174.7%, respectively, compared to 12 h), and then decreased (Figure 5a). The results of the superoxide anion removal efficiency showed that all treatment groups gradually increased over the long term (Figure 5b), although there was a slight decrease in the middle. The clearance rates of CS1, CS2, CS3, and CS4 at 72 h increased by 162.4%, 163.8%, 151.1%, and 130.8%, respectively, compared with 0 h. At the end of the storage period: CS4 < CS1 < CS2 < CS3.

### 3.4. Enzymes Activity

#### 3.4.1. Superoxide Dismutase (SOD) and Catalase (CAT) Activity

**Figure 6:**
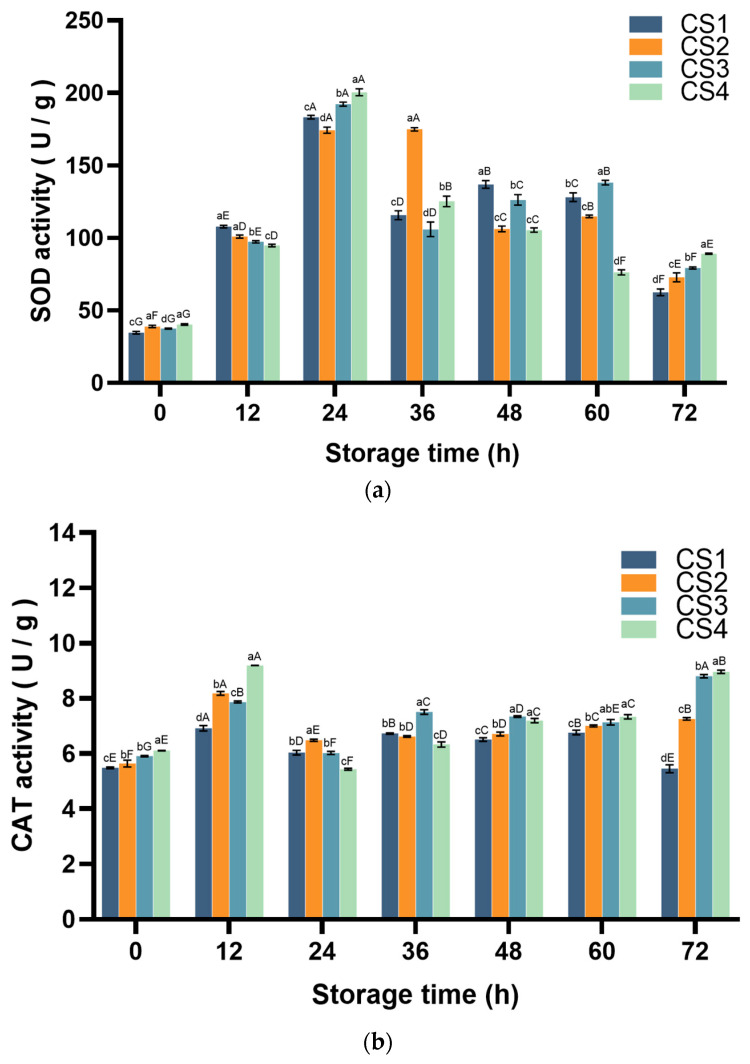
Effect of cutting method on SOD (**a**) and CAT (**b**) activity of cauliflower. Columns with vertical bars represent mean SD values (*n* = 3). Values with different letters are significantly different at *p* < 0.05. Lowercase letters represent significant differences between treatment factors; uppercase letters represent significant differences between storage times.

SOD and CAT play a crucial defensive role in the plant’s antioxidant system and are essential enzymes for fresh-cut cauliflower to resist oxidative stress. The determination of SOD is based on the generation of O_2_^−^· by xanthine and xanthine oxidase through the O_2_^−^· reaction system. SOD can reduce azatetrazole to produce blue formamide, which absorbs at 560 nm. SOD scavenges O^2−^·, thus inhibiting the formation of formazan. The darker the color of the reaction solution, the lower the activity of SOD, and vice versa, the higher it is [31]. The CAT assay is based on the ability of hydrogen peroxide to oxidize MoO_4_^2−^ to MoO_5_^2−^, which accepts the electrons from the hydroxide to form a bond, which immediately dehydrates and condenses the molecules, resulting in a stable yellow complex (H_2_MoO_4_-XH_2_O)_n_, with a strong absorption peak of 405 nm and a linear relationship between the absorbance value and the concentration of hydrogen peroxide. The changes in antioxidant enzyme system (SOD and CAT) activity are illustrated in Figure 6. For SOD activity, all treatment groups showed a continuous increase and peaked at 24 h (428.9%, 347.8%, 414.2%, and 397.2% increase from the beginning for CS1, CS2, CS3, and CS4, respectively), with CS1 decreasing at 36 h followed by a slight increase at 48 h and a subsequent decrease, and CS2 decreasing at 48 h followed by a slight increase at 60 h and a subsequent decrease. CS3 decreased at 36 h, then continued to increase until 60 h, and CS4 continued to decrease until 60 h, with a slight increase at 72 h. CAT activity, on the other hand, peaked at 12 h in all treatment groups except CS3 (26.1%, 45.1%, 33.2%, and 50.6% increase in CS1, CS2, CS3, and CS4, respectively; compared with 0 h). At 72 h, CS2, CS3, and CS4 increased by 28.7%, 49.0%, and 46.8%, respectively, compared with 0 h. The increase in CS2, CS3, and CS4 was also observed at 72 h. For each treatment group of SOD, there was a decreasing trend. At the end of the storage period: CS1 < CS2 < CS3 < CS4 (CS1, CS2, CS3 and CS4 increased by 80.4%, 87.1%, 111.8% and 120.9%, respectively, compared with 0 h).

#### 3.4.2. Ascorbate Peroxidase (APX) and Glutathione Reductase (GR) Activity

**Figure 7:**
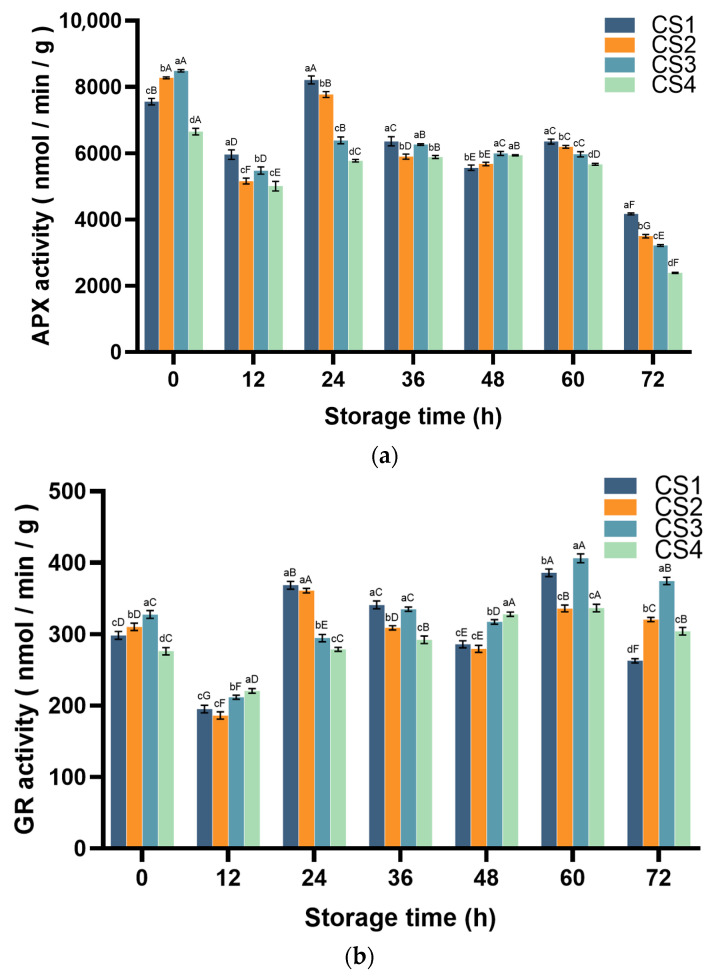
Effect of cutting method on APX (**a**) and GR activity (**b**) of cauliflower. Columns with vertical bars represent mean SD values (*n* = 3). Values with different letters are significantly different at *p* < 0.05. Lowercase letters represent significant differences between treatment factors; uppercase letters represent significant differences between storage times.

APX and GR coordinate the redox cycle mechanism of fresh-cut cauliflower and maintain cellular homeostasis by precisely controlling the ascorbate–glutathione (AsA-GSH) cycle [32,33]. GR can regenerate GSH through the NADPH-catalyzed GSSG reduction reaction, while NADPH is reduced to NADP^+^; NADPH has a characteristic absorption peak at 340 nm, whereas NADP^+^ has no absorption peak at this wavelength; therefore, the rate of NADPH reduction can be determined by measuring the rate of decrease in absorbance at 340 nm, thereby calculating GR activity [34]. APX can catalyze the oxidation of AsA by H_2_O_2_, and AsA has a characteristic absorption peak at 290 nm. The activity of APX can be calculated by measuring the rate of AsA oxidation (the rate of change in absorbance at 290 nm) [35,36]. The changes in APX and GR activity are shown in Figure 7. The main changes in APX activity were as follows: For CS1 and CS2, both continued to decline before showing a slight increase at 60 h, followed by a further decline. CS3 gradually decreased before experiencing a sharp drop at 72 h, while CS4 showed a slight increase at 36 h, followed by a decline after 48 h and a sharp drop at 72 h. At 72 h (CS1, CS2, CS3, and CS4 decreased by 44.8%, 57.7%, 62.0% and 64.1%, respectively, compared with 0 h) (Figure 7a). The changing trend in GR activity was as follows: in the first stage (0–12 h), GR decreased in all treatment groups (CS1, CS2, CS3, and CS4 decreased by 34.5%, 40.0%, 35.3 and 20.1%, respectively, compared with 0 h). The second stage (12–60 h): CS1 and CS2 both showed a decrease followed by an increase at 24 h and 60 h. CS3 continues to rise, falling at 48 h and then continuing to rise. CS4 maintained a slow increase and reached the highest level at 60 h (CS1, CS2, CS3, and CS4 increased by 29.4%, 8.3%, 24.1% and 21.9%, respectively, compared with 0 h). The third stage (60–72 h): The activity of CS1 decreased significantly (31.9%) compared to 60 h, while CS2, CS3, and CS4 decreased by 4.6%, 7.8%, and 9.6%, respectively (Figure 7b).

### 3.5. Antioxidant Capabilities

**Figure 8:**
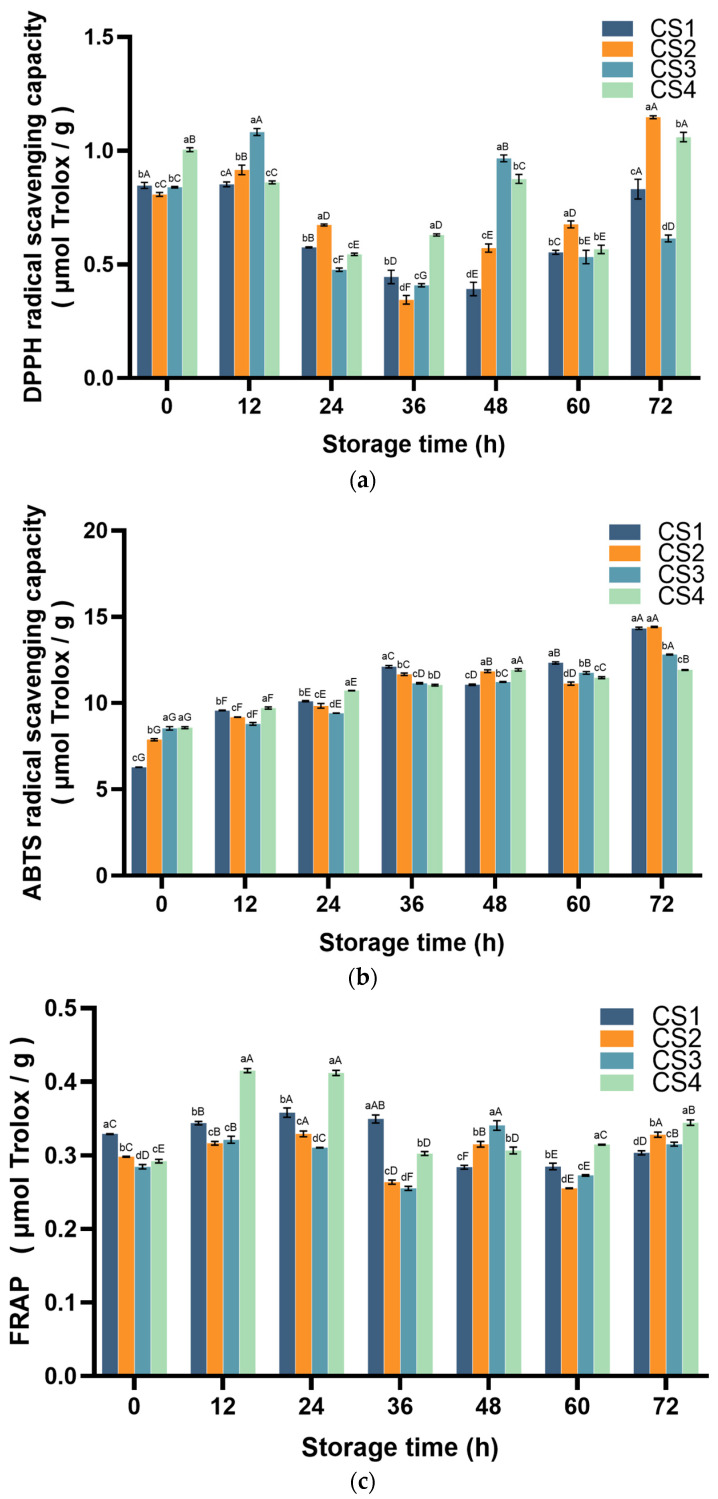
Effect of cutting method on DPPH (**a**), ABTS (**b**), and FRAP (**c**) of cauliflower. Columns with vertical bars represent mean SD values (*n* = 3). Values with different letters are significantly different at *p* < 0.05. Lowercase letters represent significant differences between treatment factors; uppercase letters represent significant differences between storage times.

The DPPH test assesses the antioxidant capacity of a sample by measuring changes in absorbance at a wavelength of 515 nm [37,38]. ABTS was determined by a free radical scavenging method using the oxidation of ABTS to produce the stable blue-green cation radical ABTS^+^·. It was measured at 734 nm and quantified by the change in absorbance [38,39]. FRAP is the ability of an antioxidant substance to reduce Fe^3+^-triphenyltriazine (Fe^3+^-TPTZ) to form blue Fe^2+^-TPTZ under acidic conditions, reflecting the total antioxidant capacity of the antioxidant substance [38,40]. The changes in the antioxidant capacity (including DPPH, ABTS, and FRAP) are shown in Figure 8. The changes in DPPH free radical scavenging activity are shown in Figure 8a. CS1 continuously decreased after 0 h, reaching its lowest level at 48 h (a 53.8% decrease compared to 0 h), and then gradually rose to return to the initial level by the end of the storage period (*p* < 0.05). CS2 levels slightly increased at the beginning of the 12 h period. Subsequently, it decreased to the lowest level at 36 h (decreased by 57.3% compared with 0 h) and then rose to the highest level from 36 h to 72 h (increased by 42.0% compared with 0 h). CS3 increased to the highest level at the beginning of 12 h (increasing by 28.8% compared to 0 h), then decreased significantly, and reached the lowest level at 36 h (decreasing by 51.4% compared to 0 h); although it increased rapidly at 48 h (increasing by 15.0% compared to 0 h), it was still lower than the initial level at the end. CS4 decreased significantly to its lowest level at 60 h (43.4% lower than at 0 h) and exhibited a trend of first decreasing and then increasing throughout the storage period, with an increase of 0.5% at the end of the storage period compared to the beginning. Unlike DPPH free radical scavenging activity, the ABTS free radical scavenging activity in all fresh-cut cauliflower tissues showed a gradually increasing trend. In terms of the degree of increase, at 72 h: CS1 > CS2 > CS3 > CS4 (increasing by 128.1%, 82.9%, 50.1%, and 38.9%, respectively, compared with 0 h) (Figure 8b). FRAP is illustrated in Figure 8c. CS1 began to rise at 36 h, decreased at 48 h, and then rebounded. While CS2 continued to rise in the first 12 h, it decreased in 36 and 60 h and then rose in 48 and 72 h. CS3 continuously decreased from 12 to 36 h, sharply increased at 48 h, and reached the highest level throughout the storage period (increasing by 19.8% compared to 0 h). CS4 reached its maximum value at 12 and 24 h of storage (increasing by 42.1% and 41.0%, respectively, compared with 0 h), decreased sharply at 36 h of storage, and gradually increased during the remaining storage periods. It can be seen from this that the cutting method has a significant impact on the antioxidant capacity of fresh-cut cauliflower. The antioxidant capacity increases with the increase in the cutting area.

### 3.6. Cauliflower Weight Loss and Color

**Figure 9:**
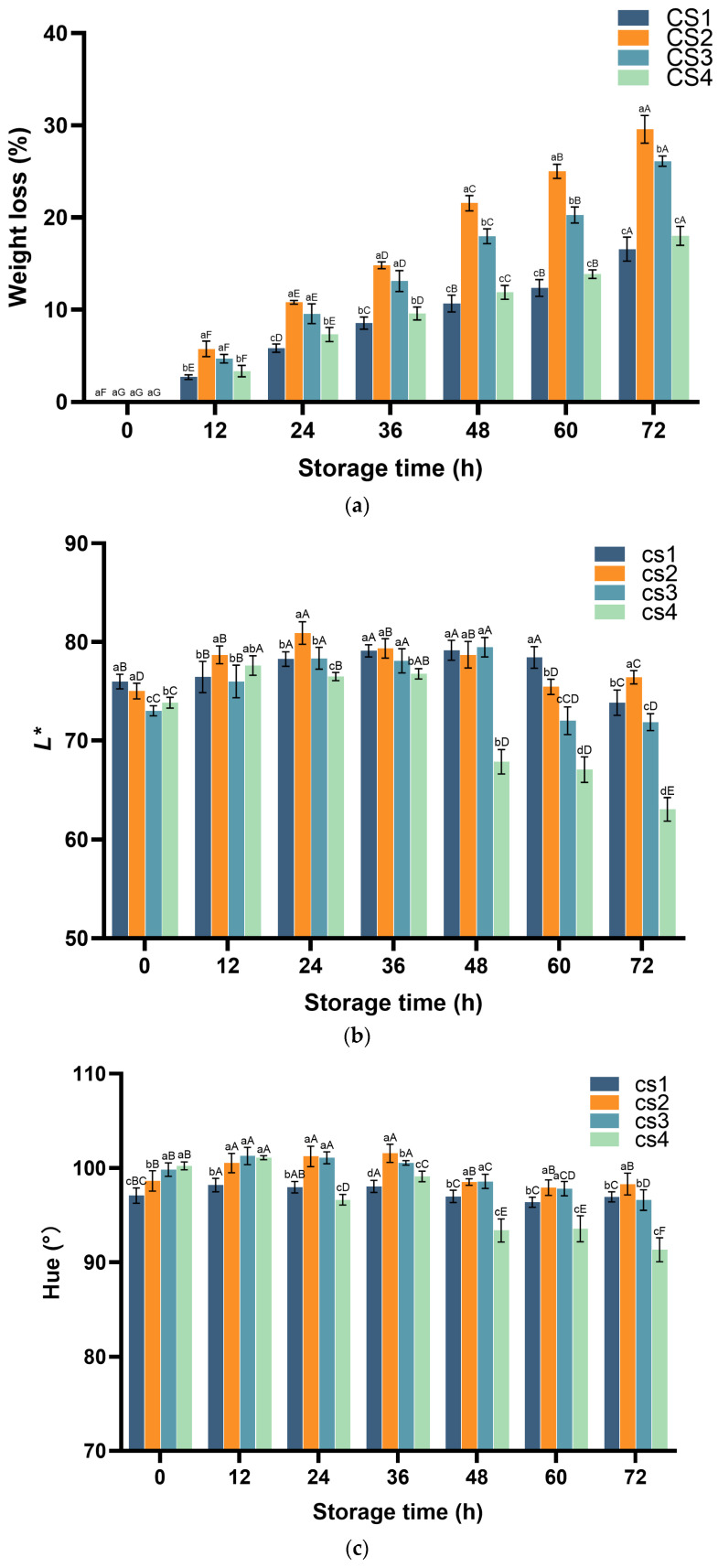
Effect of cutting method on weight loss (**a**), lightness (**b**), and WI (**c**) of cauliflower. Columns with vertical bars represent mean SD values (*n* = 3). Values with different letters are significantly different at *p* < 0.05. Lowercase letters represent significant differences between treatment factors; uppercase letters represent significant differences between storage times.

The effect of different cutting methods on weight loss in fresh-cut cauliflower samples was monitored during storage (Figure 9a). Weight loss increased significantly at each 12 h interval (*p* < 0.05) for all groups except CS1 between 48 and 60 h. As fresh-cut cauliflower loses structural integrity post-cutting, it appears more susceptible to water loss than uncut heads. CS1 exhibited lower weight loss increments than other treatments. This may stem from smaller cut surfaces limiting excessive exposure to air. Conversely, CS4 also exhibited lower increments than CS2 and CS3. This likely stems from water loss in plant samples at 15 °C storage being primarily driven by transpiration. Following chopping, CS4 experienced suppressed transpiration, consequently reducing weight loss. Color represents a key factor determining consumer acceptance of fruit quality. L* and Hue values for fresh-cut cauliflower during storage are shown in Figure 9b,c. Among these, L* values decreased significantly in all treatment groups except CS2, with CS4 experiencing a sharp decline starting at 48 h. During the 72 h storage period, L* values for all groups except CS4 remained between 70 and 80, while hue angle values ranged from 96 to 102. For CS4 samples, both L* and hue angle values stayed within these ranges from 0 to 36 h. However, after 48 h, both L* and hue angle exhibited significant decreases (*p* < 0.05), with L* falling below 68 and hue angle below 94. The hue value observed in CS4, combined with its low L* value, indicates mild browning in the vegetables. These changes are unlikely to influence consumer choice, as fresh-cut produce is typically consumed within 1–2 days of purchase.

### 3.7. Sensory Analysis

**Figure 10:**
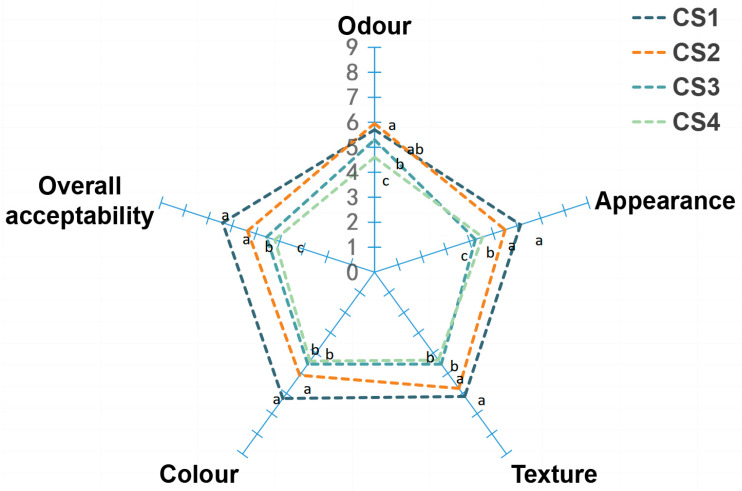
Effect of cutting methods on the sensory characteristics of cauliflower after 72 h of storage at 15 °C. The 9-point hedonic evaluation scale was used as follows: 9, like very much to 1, dislike very much. Values with different lowercases are significantly different at *p* < 0.05.

Fresh-cut cauliflower was subjected to sensory evaluation after 72 h of storage. The sensory evaluation of fresh-cut cauliflower samples with different treatments after 72 h of storage is shown in Figure 10. According to the overall acceptability score, CS1 and CS2 scored between “like a little” and “like moderately” (above 5); CS3 and CS4 scored between “dislike moderately” and “indifferent” (below 5). In odor, appearance, texture, color, only samples treated with CS1 and CS2 were acceptable (above 5). Therefore, the use of CS1 and CS2 might be ac-ceptable for further commercialization. Moreover, there is no significant difference between CS1 and CS2 for consumers (*p* > 0.05). These observations are consistent with other studies that show that cut strength versus cut style has an impact on the overall acceptability of food. For example, longitudinal cutting is more acceptable to consumers than horizontal cutting, CS2 > 5 > CS3 in terms of acceptability, and at the same time, it is difficult for consumers to accept that the cutting strength is too high, so CS3 > CS4.

## 4. Discussion

**Figure 11:**
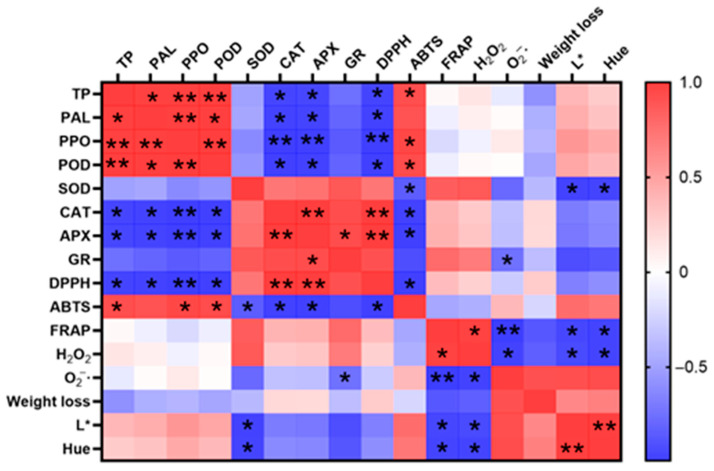
Total phenols in plants and enzymes (PPO, PAL, SOD, CAT, POD, APX, and GR) and Pearson correlation matrix of the antioxidant capacities (DPPH, ABTS, FRAP), ROS (H_2_O_2_, O_2_^−^·), weight loss and color of fresh-cut cauliflower. * represents a significant correlation at 0.05 levels. ** represents a significant correlation at 0.01 levels.

Phenolic compounds, as important antioxidant components, play a key role in maintaining the quality of fresh-cut cauliflower [8]. In this study, TP content exhibited a significant positive correlation with DPPH antioxidant capacity (*p* < 0.05), consistent with the findings of previous studies on barley malt and kiwifruit [10,41]. Additionally, CS3 and CS4 significantly outperformed CS1 and CS2 in enhancing TP content (*p* < 0.05). Furthermore, during the later stages of storage, higher cutting intensity resulted in greater TP accumulation. The same results were found in fresh-cut carrots and mushrooms [42,43]. The ABTS results showed that the total reduction capacity of all fresh-cut cauliflower treatment groups was always improving, and CS1 and CS2 were higher than those of other treatment groups at 72 h. ABTS was able to detect the overall ability of samples to provide electrons to neutralize free radicals, and could detect both water-soluble and fat-soluble antioxidant capacity in samples, indicating that CS1 and CS2 cauliflower produced more water- and fat-soluble antioxidants after cutting [38,44]. In the FRAP results, the total reducing power of all fresh-cut cauliflower treatment groups was significantly increased except for the last decrease in CS1 (*p* < 0.05). The difference from other antioxidant capacity results may be due to the fact that FRAP is detected by pure single electron transfer, while glutathione and some proteins in the sample cannot be detected by this method. Figure 11 shows that ABTS and DPPH were significantly correlated with the activities of TP, PPO, POD, CAT, and APX in fresh-cut cauliflower samples (*p* < 0.05), while FRAP was significantly associated with ROS and color. The effects of FRAP and ROS may be related to the AsA-GSH cycle, which is also seen in similar broccoli [45]. According to existing research, the increase in antioxidant capacity is primarily attributed to the accumulation of phenolic compounds. Broccoli contains various phenolic compounds, including hydroxycinnamic acids (caffeic acid, ferulic acid, octanoic acid, and p-coumaric acid), flavonoids (including quercetin, kaempferol, and isorhamnetin), and anthocyanins (acetylated anthocyanins), such as anthocyanin-3-mountain ash acid-5-glucoside, lignans, and other compounds [46]. Damage stimuli induce phenolic synthesis, further increasing total phenolic content, a phenomenon also observed in broccoli [4,47]. In plants, the accumulation of phenolic compounds depends on the dynamic relationship between phenolic synthesis and oxidative reactions [48], which is the primary reason for the inconsistent trends in phenolic compound content in fresh-cut cauliflower. Phenolic compounds, as important secondary metabolites, are synthesized through the phenylpropanoid metabolic pathway [6].

PAL is the first key enzyme in the phenylpropanoid metabolic pathway. It catalyzes the conversion of phenylpropyl to trans-cinnamic acid and further to phenolics such as caffeic acid and coumaric acid [49,50]. In the present study, fresh-cutting induces PAL activity at an earlier storage period (Figure 3b), which accelerates the rate of phenol synthesis. Similarly to total phenol content, the effect of PAL activity on wound type was significant and increased with the intensity of the wound, similar to the results in broccoli [4]. In the present study, TP content showed a significant positive correlation with PAL activity (*r* = 0.977, *p* < 0.01) (Figure 9). In the present study, PAL activity was observed to be CS1 < CS2 = CS3 < CS4 at 0 h. In the PAL results, the activities of both CS1 and CS3 recovered to their initial levels by the end of the storage period, while the activities of both CS2 and CS4 increased up to 24 h prior to the end of the storage period and then began to decrease. Although the activity recovered at the end of the storage period, it remained significantly below the initial level at the beginning of the storage period. This suggests that PAL activity is also affected by the direction and intensity of cutting, a phenomenon also seen in fruits and vegetables such as pineapple [51].

PPO, as a copper-containing oxidoreductase, can catalyze the oxidation of phenolic compounds into quinones. In this process, the phenolics, which serve as substrates for polyphenol oxidase, undergo oxidation catalyzed by the enzyme [52]. The change in PPO activity was significantly different from the cutting method. At the end of the storage period, the PPO activities of CS1 and CS2 decreased significantly, while those of CS3 and CS4 increased and were nearly equal, being significantly higher than those of the first two groups. A highly significant positive correlation was found between the PPO activities and the TP content (*r* = 0.981, *p* < 0.01). This may be because many phenolic compounds from vegetables possess ortho-dihydroxy groups, which can serve as potential specific substrates for PPO [53]. In contrast, POD activity showed a change in CS2 and CS3 > CS4 at 72 h. This antagonistic effect of POD activity may be due to CS4 losing moisture and releasing polyphenols at the end of storage, thereby enhancing antioxidant capacity and reducing polyphenol oxidase activity [54,55].

For ROS, H_2_O_2_ levels peaked between 24 and 36 h in all treatment groups, with the CS4 treatment group showing significantly higher cumulative H_2_O_2_ levels than the other groups (*p* < 0.05). Unlike the changes in the content of H_2_O_2_, the O_2_^−^· scavenging capacity in fresh-cut cauliflower was immediately induced by the cutting process; similar results were also observed in various products such as kiwifruit [10], indicating that fresh-cut processing leads to excessive oxidative stress. Furthermore, this study demonstrates that the O^2−^· scavenging capacity increases with the severity of injury. This may be because as the intensity of the wound increases, a larger wound surface becomes exposed to O_2_^−^·, accelerating the peroxidation process and leading to the accumulation of O_2_^−^·, a phenomenon also documented in studies on fresh-cut pumpkin [56].

SOD, POD, and CAT are natural scavengers of O_2_^−^· and H_2_O_2_, catalyzing the degradation of ROS into H_2_O and O_2_. The AsA-GSH cycle interacts with ROS in a non-enzymatic manner [57]. The AsA-GSH cycle relies on APX and GR to regulate AsA and GSH metabolism [58]. APX uses AsA to detoxify H_2_O_2_, producing MDA, a radical intermediate that enhances stress tolerance. MDA can be recycled back to AsA or oxidized to DHA, which is then reduced to AsA by DHAR and GSH [59]. In the present study, as APX catalyzes the scavenging of free radicals by MDA, its content decreases gradually during the storage of fresh-cut cauliflower. This phenomenon is consistent with previous reports [60]. With the decrease in ASA, the obtained DHAR can synthesize GSH via GR, thus providing an additional substrate for GSH synthesis. This explains the rise in GR activity during late storage. In this study, the correlation coefficient between APX and DPPH was 0.998 (*p* < 0.01) (Figure 9), suggesting that cauliflower can be subjected to cutting damage by modulating APX activity, which in turn improves the antioxidant capacity of fresh-cut cauliflower. In the antioxidant enzyme system, SOD, POD, and CAT are natural scavengers of O_2_^−^· and H_2_O_2_, catalyzing the decomposition of ROS into H_2_O and O_2_. Enzymes such as SOD, CAT, and APX are significantly correlated with antioxidant capacity. In this study, antioxidant enzymes (SOD, POD, CAT, and APX) exhibited strong correlations with antioxidant capacity (r = −0.959, 0.911, 0.997, and 0.998), respectively (Figure 9). These results suggest that regulating antioxidant enzyme activity and superoxide anion content can enhance the antioxidant capacity of fresh-cut cauliflower through the cutting process, thereby improving its antioxidant properties over time.

## 5. Conclusions

Our findings reveal a relationship between the initial intensity and sustained duration of antioxidant responses in freshly cut cauliflower, which is directly influenced by the method of cutting. The findings demonstrated a significant positive correlation between the cutting technique and antioxidant capacity. Transverse cutting markedly promoted phenolic compound accumulation and enhanced reactive oxygen species (ROS) scavenging within the short term (0–48 h). However, over an extended period (48–72 h), it led to a decline in enzyme activity—particularly the collapse of APX—attributable to intensified oxidative stress. In contrast, longitudinal cutting sustained a more stable level of antioxidant activity. Throughout this period, antioxidant enzymes, including POD, CAT, and APX, played essential roles and exhibited strong correlations with overall antioxidant capacity. Therefore, the choice of cutting method is essentially a choice between inducing high but transient antioxidant peaks and achieving a moderate but sustained antioxidant boost, combined with the preferences of market consumers. Based on the aforementioned research findings, it is recommended to employ a longitudinal slicing technique to balance phenolic compound induction and oxidative damage, thereby prolonging the duration of antioxidant activity while reducing moisture loss and quality deterioration. This discovery provides crucial theoretical groundwork for developing postharvest preservation technologies for fresh-cut cauliflower.

## Data Availability

The original contributions presented in this study are included in the article. Further inquiries can be directed to the corresponding authors.
